# Composites of Unsaturated Polyester Resins with Microcrystalline Cellulose and Its Derivatives

**DOI:** 10.3390/ma13010062

**Published:** 2019-12-21

**Authors:** Artur Chabros, Barbara Gawdzik, Beata Podkościelna, Marta Goliszek, Przemysław Pączkowski

**Affiliations:** Department of Polymer Chemistry, Institute of Chemical Sciences, Faculty of Chemistry, Maria Curie-Sklodowska University in Lublin, M. Curie-Sklodowska Sq. 5, 20-031 Lublin, Poland; barbara.gawdzik@poczta.umcs.lublin.pl (B.G.); beatapod@poczta.umcs.lublin.pl (B.P.); marta.goliszek@poczta.umcs.lublin.pl (M.G.); przemyslaw.paczkowski@poczta.umcs.lublin.pl (P.P.)

**Keywords:** microcrystalline cellulose (MCC), composites, unsaturated polyester resins, thermogravimetric analysis (TG), mechanical analysis, dynamic mechanical analysis (DMA)

## Abstract

The paper investigates the properties of unsaturated polyester resins and microcrystalline cellulose (MCC) composites. The influence of MCC modification on mechanical, thermomechanical, and thermal properties of obtained materials was discussed. In order to reduce the hydrophilic character of the MCC surface, it was subjected to esterification with the methacrylic anhydride. This resulted in hydroxyl groups blocking and, additionally, the introduction of unsaturated bonds into its structure, which could participate in copolymerization with the curing resin. Composites of varying amounts of cellulose as a filler were obtained from modified MCC and unmodified (comparative) MCC. The modification of MCC resulted in obtaining composites characterized by greater flexural strength and strain at break compared with the analogous composites based on the unmodified MCC.

## 1. Introduction

The pressure exerted by the increase of public awareness to limit human interference in the environment affects also trends in polymer chemistry development. Adaptation of raw materials from renewable resources to the existing technological solutions is one way to achieve this goal [1,2,3,4]. The main factor determining the above activities is the renewable nature of the raw material base; easy availability; and, above all, its biodegradability [5,6].

One group are biopolymers, where cellulose is the main representative. Cellulose modifications and preparation of composites are extensively studied in the literature [7,8,9]. Zadorecki and Flodin [10] investigated the effects of interfacial adhesion of cellulose–polyester composites on their environmental aging behavior. The authors observed that, when the matrix and the cellulose fiber are covalently bonded, no cracks are formed during shrinking. In the other work [11], these authors studied the reinforcement effect of cellulose fibers treated with different coupling agents on unsaturated polyesters. Abdelmouleh et al. [12] tested mechanical properties of unsaturated epoxy and polyester resin matrices filled with silane-treated cellulose fibers. Their reinforcing effect proved to be promising, especially with some silane-treated fibers, and improvement was especially visible for methacrylic silane in contact with an unsaturated polyester resin. Rodrigues et al. [13] studied the sugarcane bagasse fibers modified by estherification to use as reinforcement in a polyester matrix. The authors proved that composites were characterized by better mechanical strength compared with pure polymer. DiLoreto et al. [14] developed a preliminary process for incorporating freeze-dried cellulose nanocrystals powder into polyester resin, where functionalized cellulose particles were used as a potential reinforcement for an unsaturated polyester resin system, a common material for automotive applications. Kargarzadeh et al. [15] prepared new nanocomposites with unsaturated polyester resin (UPR) and silane surface-treated cellulose nanocrystals. Tensile tests showed that both the stiffness and strength of the UPR improved upon the incorporation of cellulose. 

Its attractiveness for use is because of the fact that it is an easily available material obtained on a large scale in the paper industry [16]. An important commercial product is also its microcrystalline form obtained by partial depolymerization of cellulose, most often as a result of acid hydrolysis. In this form, it found a number of applications, including the production of medicines, cosmetics, and food, where it mostly acts as a filler [17]. The use of microcrystalline cellulose (MCC) of the same nature was also of significant interest in the preparation of composites based on generally available synthetic polymers [18,19].

However, obtaining homogeneous composites creates problems owing to the hydrophilic nature of the cellulose surface. This is not compatible with the hydrophobic polymer matrix. Uniformity of the filler/matrix surface has a major impact on the load carrying capacity, and thus on the properties of composites. This effect can be achieved using compatibilizers that reduce the tension between phases [20] or modifying the cellulose surface to obtain a less hydrophilic one [21]. Surface modification also allows introducing unsaturated bonds to the filler structure. The development of its surface also has a significant impact on the ability to transfer loads. The combination of this procedure and the use of unsaturated polyester resin as a matrix and nanocellulose modified with unsaturated fatty acids allowed to improve the mechanical properties of composites through the copolymerization of their individual components expressed as increased tensile strength [22]. However, the use of the fillers at nanometer scale in mixtures with resins has limitations. The development of their surface causes the dispersion of the particles, which is accompanied by absorption of the polymer matrix, which causes the viscosity of the systems to increase even at low load, which prevents their practical use [23].

Furthermore, reduction of cellulose particle sizes is associated with additional procedures and costs [24], whereas the microcrystalline form of cellulose is much cheaper and readily available.

The aim of this research was to improve the miscibility of MCC in the unsaturated polyester resin. This effect was obtained by modifying the surface of its microcrystalline form using methacrylic anhydride. The presence of unsaturated bonds additionally allowed the chemical binding of the filler to the structure of the polymer matrix network. In order to develop the filler/matrix interface between the phases and skip the procedure of obtaining cellulose on the nano scale, the decision was made to increase the load on the resin with the filler.

## 2. Materials and Methods

### 2.1. Chemicals

The unsaturated polyester resin (UPR) used for the study consisted of 70% of unsaturated polyester and 30% of styrene as a crosslinking monomer. The detailed description of this synthesis and curing procedure was presented in the work of [25].

To modify MCC (Chem Point, Kraków, Poland) pyridine and hydroquinone (Avantor, Gliwice, Poland), 4- (Dimethylamino) pyridine (DMAP) and methacrylic anhydride (Sigma-Aldrich, Saint Louis, MO, USA) were used.

#### 2.1.1. Modification of microcrystalline cellulose MCC

First, 50 g of MCC, 1000 cm^3^ of pyridine, 12.5 g of DMAP as catalyst, and 6.9 g of hydroquinone as inhibitor were placed in a 2000 mL flask. The reagents were heated to 80 °C and constantly stirred. Next, 314 cm^3^ of methacrylic anhydride was added dropwise over 30 min. Heating at 100 °C was continued for 2 h. The obtained product was filtered under the reduced pressure. The precipitate was washed with water and then acetone. The modified MCC was dried in the air at 40 °C to constant weight.

#### 2.1.2. Preparation of Composites

To the unsaturated polyester resin (UPR), which was pre-accelerated with 0.45 wt% of the cobalt octoate solution and 1.2 wt% of the DMPT solution (N,N-dimethyl-*p*-toluidine, 10% solution in styrene), 2 wt%, 5 wt%, and 10 wt% of modified MCC (CEL-MOD) was added, respectively. For homogeneity, it was mixed and then placed in an ultrasonic bath for 15 min. This procedure was repeated twice. To this mixture, 3 wt% of Luperox DHD-9 (the methyl ethyl ketone peroxide solution) as an initiator was added. Its amount was calculated for the resin. The prepared mixtures were poured into cuboid-shaped molds with dimensions 14.5 cm × 10 cm × 0.4 cm. Curing was conducted in the molds at room temperature for 24 h. Then, for additional post-curing, the moldings were placed in an oven for 10 h at 90 °C. The same procedure was applied for the composites of unmodified MCC (CEL-UNMOD) and pure resin (UPR).

### 2.2. Research Methods

Attenuated total reflectance Fourier transform infrared (ATR-FTIR) spectra were obtained with a Bruker TENSOR 27 spectrometer with a diamond crystal (Ettlingen, Germany). The spectra were gathered from 600 to 4000 cm^−1^ with 32 scans per spectrum at a resolution of 4 cm^−1^.

Mechanical studies were carried out using the mechanical testing machine, ZwickRoell Z010 (Ulm, Germany). With a three-point bending test, the 80 mm × 10 mm × 4 mm sample profiles were used. The support spacing was 64 mm, whereas the bending speed was 5 mm min^−1^. The final result was the arithmetic averaging of five measurements.

Hardness was determined using a hardness tester HPK (Leipzig, German Democratic Republic) and the Brinell method was applied. The final result was the mean value of 10 measurements.

Thermomechanical properties of materials were determined using the dynamic mechanical analyzer (DMA) Q800 from TA Instruments (New Castle, DE, USA) equipped with a double-cantilever device. The samples of 65 mm × 10 mm× 4 mm dimension were tested. The temperature scanning from 0 to 200 °C with a constant heating rate of 3 °C min^−1^ at a sinusoidal distortion of 10 µm amplitude and 1 Hz frequency was conducted. The glass-transition temperature, damping factor, values of storage modulus, and loss modulus were determined.

Thermal resistance of the obtained materials was measured by the thermogravimetric analysis (TG/DTG) using a NETZSCH STA 449 Jupiter F1 TG analyzer (Selb, Germany), Al_2_O_3_ crucibles, and the sample mass ca. 10 mg. The analyses were performed in the helium atmosphere with the flow rate of 20 mL min^−1^ in the temperature range of 35–800 °C and the heating rate of 10 °C min^−1^.

The differential scanning calorimetry (DSC) analyses were performed on a Netzsch DSC 204 calorimeter (Selb, Germany). The scans were carried out in a nitrogen atmosphere (30 cm^3^ min^−1^) at a heating rate of 10 °C min^−1^. The mass of the sample was approximately ~7 mg. As a reference, an empty aluminum crucible was used.

## 3. Results and Discussion

### 3.1. Attenuated total reflectance Fourier transform infrared spectroscopy analysis ATR/FTIR

**Figure 1:**
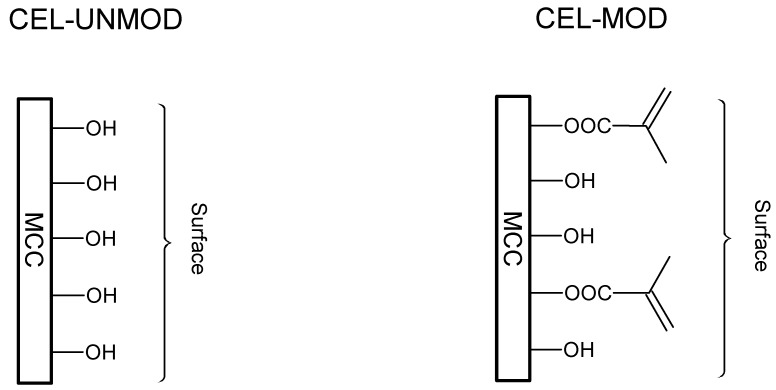
Schematic representation of unmodified microcrystalline cellulose (MCC) (CEL-UNMOD) and modified MCC (CEL-MOD) surface.

**Figure 2:**
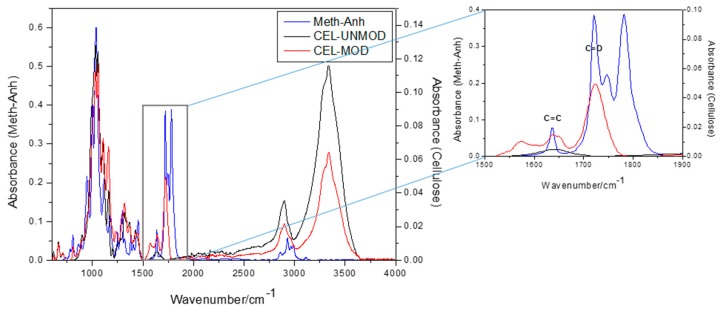
Attenuated total reflectance Fourier transform infrared (ATR/FTIR) spectra of methacrylic anhydride (Meth-Anh) and MCC before and after modification.

The MCC used for the preparation of composites was first subjected to the ATR/FTIR analysis. To follow the explanations on the molecular level, the schematic representation of MCC before modification (CEL-UNMOD) and its methacrylic derivative (CEL-MOD) are presented in Figure 1. Figure 2 shows the spectra of methacrylic anhydride (Meth-Anh), CEL-UNMOD, and CEL-MOD. The efficiency of MCC esterification with methacrylic anhydride is generally demonstrated by three ranges: the peak at 1723 cm^−1^ is attributed to the stretching vibrations of C=O carbonyl groups, indicating an ester bond formation. The fuzzy band observed at 1638 cm^−1^ in the spectrum of CEL-UNMOD is connected with the presence of water adsorbed by the crystalline part of cellulose [26]. Its intensity increases after esterification, which may indicate overlapping by the signal of the C=C stretching vibrations from methacrylic groups. A wide peak in the range of 3800–3300 cm^−1^ is associated with the stretching vibrations of hydroxyl groups. The presence of this peak with reduced intensity in the case of CEL-MOD indicates that not all hydroxyl groups were blocked and the esterification process was incomplete. The degree of substitution (DS) = 0.02 was estimated alkacimetrically according to the procedure presented in the work of [27].

**Figure 3:**
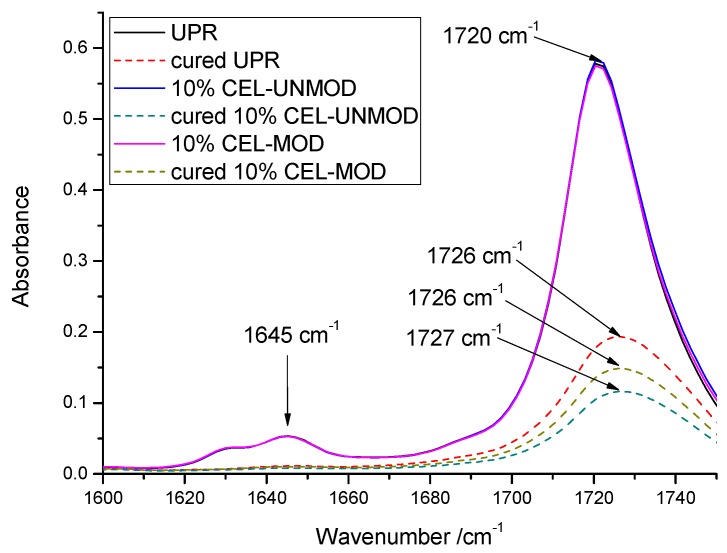
ATR/FTIR spectra of unsaturated polyester resin (UPR) and composites with 10% CEL-MOD/UNMOD before and after curing.

UPR and its systems containing 10% filler—both before and after curing—were also subjected to ATR/FTIR analysis. Despite the slight differences in the wide absorption spectrum, discernible differences occur in the range of 1750–1600 cm^–1^ shown in Figure 3. The main point is the disappearance of the peak at 1645 cm^−1^ corresponding to stretching C=C, confirming the effectiveness of the curing process. In addition, the lack of this peak in the case of a composite with 10% CEL-MOD may suggest that, in the heterogeneous curing of the polyester matrix [28], the unsaturated bond of the methacrylic group to CEL-MOD may also be involved. Shifts of signals from the C=O carbonyl group have also been observed. They were related to the intermolecular interaction of hydrogen bonds with hydroxyl groups. The presence of a signal at a higher wavelength for a CEL-UNMOD composite of 1727 cm^–1^ compared with a CEL-MOD composite of 1726 cm^−1^ indicates greater matrix–filler interaction through hydrogen bonds for CEL-UNMOD composites [29,30].

### 3.2. Mechanical Tests

**Figure 4:**
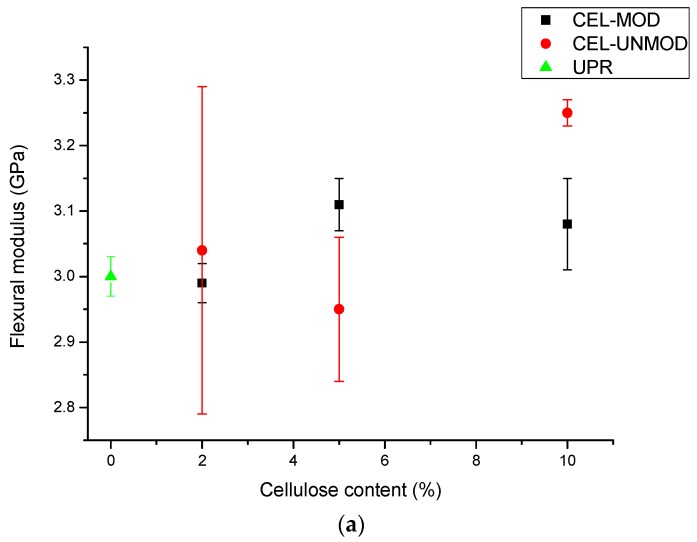

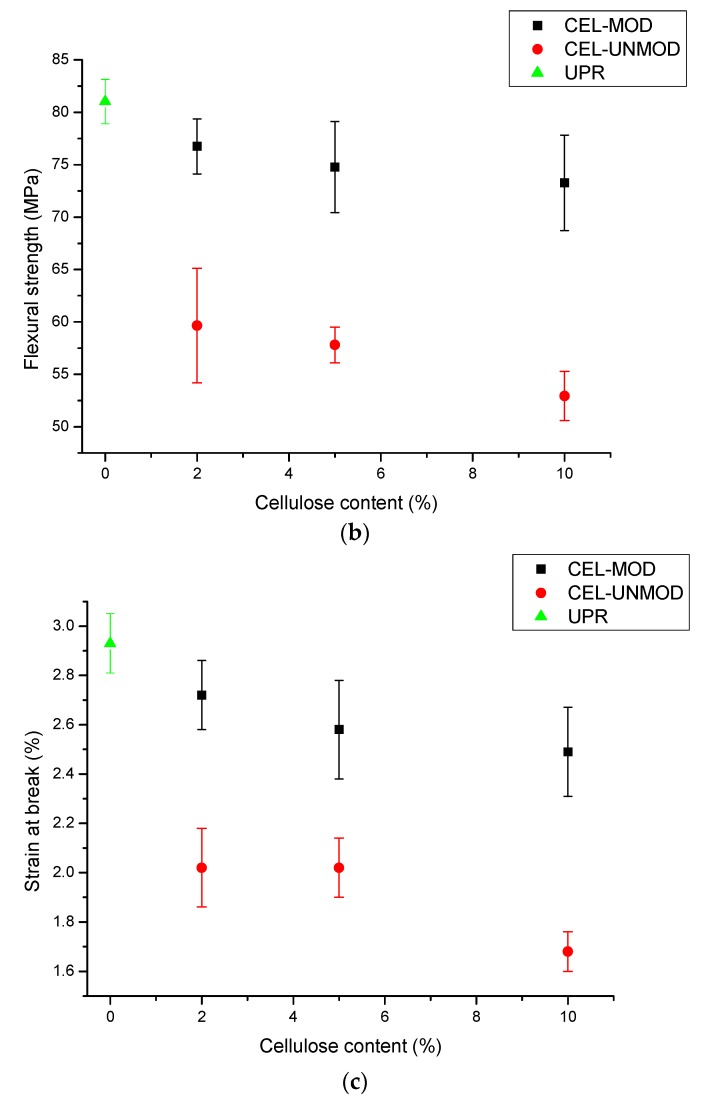
Mechanical data of composites and UPR. (**a**) flexural modulus; (**b**) flexural strength; (**c**) strain at break.

The mechanical properties of pure resin (UPR) and composites containing unmodified and modified MCC were determined. The results of flexural strength tests are presented graphically in Figure 4 and the numerical data of the flexural modulus, flexural strength, and strain at break are collected in Table 1.

In both the CEL-MOD and CEL-UNMOD systems, an increase in the flexural modulus value is visible as the filler content increases. This is justified by the increased loading of composites by the crystalline phase present in the cellulose structure. However, in the case of systems with CEL-UNMOD, it is higher than for analogous systems with CEL-MOD. This would confirm that the decisive factor affecting its value is the interactions of matrix intermolecular hydrogen bonds (polyester carbonyl groups) with MCC hydroxyl groups. This is particularly visible for systems containing 10% filler. The addition of 5% of CEL-UNMOD reduces the value of the flexural modulus compared with that of pure resin. This can be attributed to the heterogeneous distribution of the filler in the matrix. The low compatibility of the hydrophilic character of the MCC surface with the hydrophobic nature of the resin leads to a decrease in the filler dispersion in the system through the formation of agglomerates [31]. Increasing the content of CEL-UNMOD to 10% makes the clusters’ formation more difficult [32]. Comparing the composites containing 5% of the filler, a higher flexural modulus is observed for the system with CEL-MOD. Increasing the hydrophobic character through modification increases the filler compatibility with the matrix. The increase in homogeneity of the CEL-MOD-based composites in comparison with those of CEL-UNMOD indicates that they are characterized by higher flexural strength values as well as higher values of strain at break. The composites with CEL-MOD are less brittle compared with the analogous ones based on CEL-UNMOD. However, the fragility of composites with both CEL-MOD and CEL-UNMOD increases with the increasing amount of filler.

It seems that the hardness of the composites will depend only on the loading of the resin with the filler. The results of the hardness test, presented in Table 1, however, indicate that an upward tendency compared with the cured resin was observed only for composites with CEL-MOD. The hardness of the CEL-UNMOD systems shows some analogy with the values of the flexural modulus. For systems containing 2% and 5%, they are lower than for UPR. In addition, the system with 5% filler is characterized by the lowest hardness value. This relation corresponds well with the results of other studies, thus determining the effect of MCC modification on the quality of the filler dispersion in the matrix.

**Figure 5:**
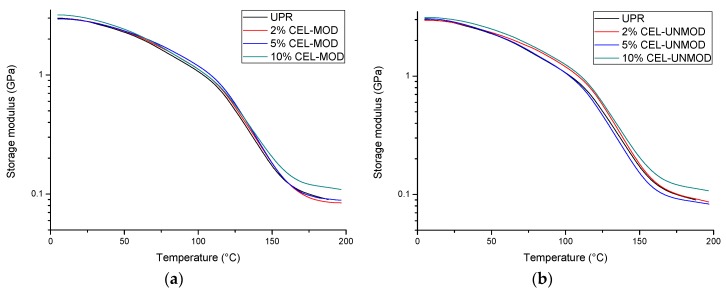
Storage modulus (E’) of UPR and composites with CEL-MOD and CEL-UNMOD. (**a**) CEL-MOD; (**b**) CEL-UNMOD.

**Figure 6:**
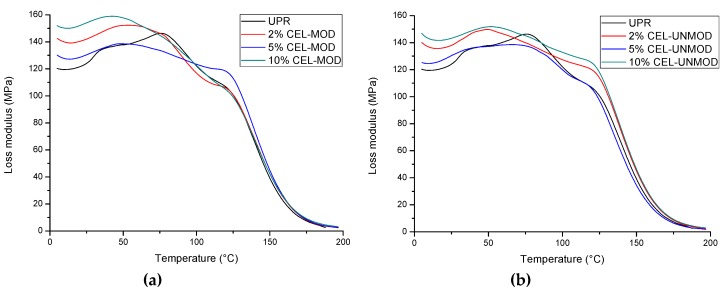
Loss modulus (E”) of UPR and composites with CEL-MOD and CEL-UNMOD. (**a**) CEL-MOD; (**b**) CEL-UNMOD.

**Figure 7:**
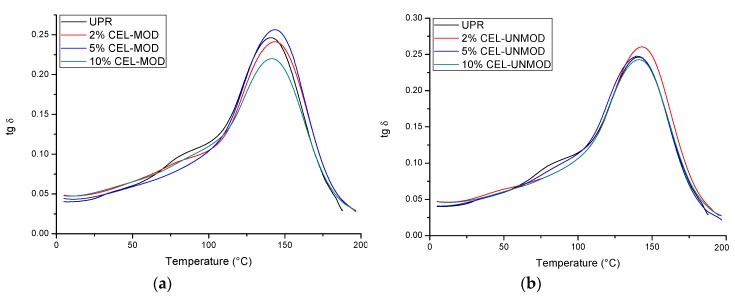
Damping factor (tg δ) of UPR and composites with CEL-MOD and CEL-UNMOD. (**a**) CEL-MOD; (**b**) CEL-UNMOD.

The results of dynamic-mechanical analysis (DMA) in a wide temperature range allowed for a more detailed assessment of the effect modified MCC (CEL-MOD) on the composites properties. The changes of the storage modulus (E’) (Figure 5), loss modulus (E”) (Figure 6), as well as the damping factor (tan δ) (Figure 7) are presented. The numerical values of the storage modulus (in the glassy and rubbery regions), glass transition temperature (read with the maximum tg δ), degree of heterogeneity (FWHM, full width at half maximum), and values of the damping factor are presented in Table 1.

In the composites with CEL-UNMOD, the storage modulus in the glassy region E’(25 °C) increases with the increasing amount of filler. This may indicate that the rigidity of the material is mainly owing to the resin loading with the filler. Despite the increase in the filler amount, no modulus changes are observed for 2% and 5% CEL-MOD in comparison with pure resin. This difference may result from the fact that the material stiffness is additionally determined by the filler/matrix interphase interactions. The introduction of groups more compatible with the hydrophobic nature of the resin onto the MCC surface would contribute to the plasticization of the system [33]. However, for the mixtures containing 10% of the filler (CEL-MOD and CEL-UNMOD), the modulus values are the highest and converge. This would suggest that, in this case, the CEL-MOD plasticizing effect is levelled by the dominant influence of the filler amount. The above assumptions are in good agreement with the storage modulus of composites in the rubbery region E’(180 °C). This is related to the material ability to carry loads resulting from the restriction of the movement of matrix polymer chains. The increase in the modulus value as the filler content increases in the systems with CEL-MOD and CEL-UNMOD shows that this movement is more and more difficult. However, a decrease in the modulus value for 5% of CEL-UNMOD would confirm the dominant effect of the heterogeneous dispersion of the filler in the resin. In contrast, lower modulus values for 2% and 5% of CEL-MOD systems in the comparison with pure resin would indicate the overwhelming effect of the plasticizing associated with reduced friction at the filler/matrix interface.

The loss modulus E" (Figure 6) allowed to assess the ability of composites to dissipate energy during deformation in the form of heat, and thus to determine their viscous response. The matrix and filler consistency has a significant impact on it [34]. The maximum modulus values for the composites with CEL-MOD are higher than for the analogous systems with CEL-UNMOD. This may indicate that the unsaturated bonds present in CEL-MOD are embedded in the polymer structure of the matrix. Large values of the loss modulus E" in a wide temperature range indicate the heterogeneity of the structure of the tested materials. However, determination of their degree in full width at the half maximum (FWHM) (Table 1) from the damping factor tg δ (Figure 7) showed insignificant changes. It can thus be assumed that this is associated mainly with the heterogeneous structure of the polymer matrix network visible in the form of two peaks on the tg δ curve. This results from the ability to copolymerize UPR components and the formation of microgels during its curing [35]. However, the addition of filler reduces the damping capacity in the range of 60–110 °C. This may suggest that it limits the formation of microgel clusters and reduces the heterogeneity of the polymer matrix network. In addition, when CEL-MOD is used, a decrease in the damping ability in this temperature range can confirm participation of the double bonds from the filler in the polymer network formation. The consistency of the matrix with the filler affects the glass transition temperature. The highest T_g_ values were found in 2% of CEL-UNMOD and 5% of CEL-MOD systems.

### 3.3. Thermal Analysis

**Figure 8:**
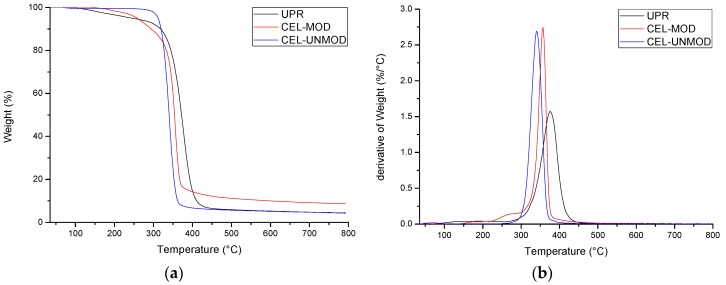
Thermograms (TG/DTG) of composite components. (**a**) TG; (**b**) DTG.

**Figure 9:**
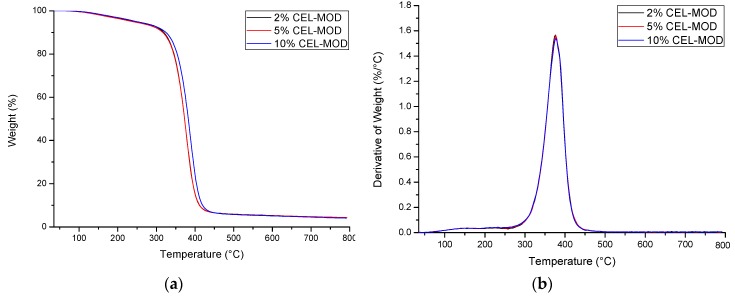
Thermograms (TG/DTG) of composites with CEL-MOD. (**a**) TG; (**b**) DTG.

**Figure 10:**
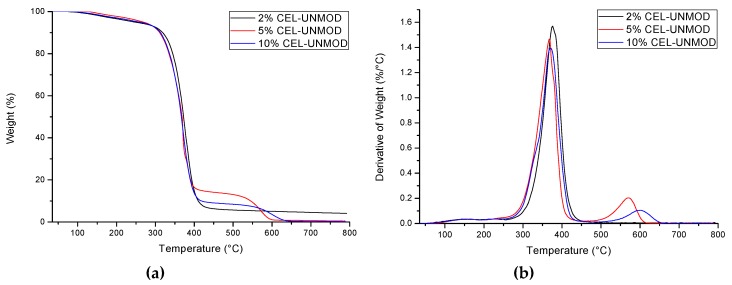
Thermograms (TG/DTG) of composites with CEL-UNMOD. (**a**) TG; (**b**) DTG.

Thermogravimetric studies allowed to determine the thermal stability of composites. Characterization of the distribution of individual components of the mixtures (UPR, CEL-MOD, and CEL-UNMOD) (Figure 8) and the systems with CEL-MOD (Figure 9) and CEL-UNMOD (Figure 10) allowed to determine the interactions of the filler with the matrix. The numerical data of thermal analysis are presented in Table 2.

CEL-UNMOD decomposition takes place in one temperature range of ca. 275–400 °C with a maximum decomposition temperature T_max1_ = 340.5 °C. This is attributed to one mechanism by which its degradation proceeds. In the case of CEL-MOD, in addition to the main decomposition, whose maximum occurs at T_max1_ = 356.6 °C, there is an initial step mass loss. The first one in the range of ca. 120–220 °C is associated with the moisture present in the MCC structure, while the second one in the range of ca. 220–300 °C can be attributed to the decomposition of methacrylic groups. The main UPR decomposition associated with the degradation of the spatial structure of the polymer network occurring in the range of about 280–450 °C is preceded by a mass loss in the range of ca. 100–280 °C, associated with the evaporation of unreacted substrates of the synthesis of unsaturated polyester and styrene that were not incorporated into the polymer network during UPR curing and low-boiling and weakly connected components.

The presence of a filler in the resin affects the temperature of the main decomposition of the system. In the case of the composites with CEL-UNMOD, T_max1_ increases for the system with 2% of CEL-UNMOD, whereas 5% and 10% contents of the filler reduce it below the maximum decomposition temperature of pure resin. These changes are in good agreement with the results obtained from the mechanical and thermomechanical tests, and can be related to the degree of CEL-UNMOD dispersion in the matrix. The heterogeneity of the filler distribution can also be reflected in the mass loss for the above systems in the range of about 500–650 °C with T_max2_ = 568.2 °C for 5% of CEL-UNMOD and T_max2_ = 595.2 °C for 10% of CEL-UNMOD. Agglomerates can decompose at higher temperatures as a result of the protective layer formation in the form of carbon derived mainly from the resin pyrolysis. In the CEL-MOD systems, the maximum decomposition temperature T_max1_ increases with the increasing filler content. This can confirm the improved MCC miscibility obtained through modification. In addition, the T_max1_ values, which are higher for composites than for individual components, would indicate a difficult decomposition of the systems resulting from the incorporation of MCC through unsaturated bonds of methacrylic groups in the structure of the polymer matrix network. In addition, the effect of the filler interactions with the resin is also visible in the mass loss of composites compared with its individual components in the range of 220–300 °C. The observed differences in the mass loss can be attributed to the formation of clusters of polymer networks of different compositions in the mixtures. This would correspond to the theory of microgel formation during curing of the resin. For the 2% CEL-UNMOD system, a 0.10% decrease in mass loss could indicate a reduction in its formation. However, a higher CEL-UNMOD resin load results in an increase in mass loss. The maximum is for the 5% CEL-UNMOD one, being 1.58%. The ability to agglomerate the filler particles would thus facilitate the formation of areas of the polymer network that are more easily degraded. In the case of CEL-MOD, the largest difference in mass loss occurs for the system containing 2% of the filler, amounting to 0.23%. The increase in the CEL-MOD resin loading results in a decrease in the mass loss difference. For the 10% CEL-MOD system, the total mass loss of individual components is the same. Therefore, it can be assumed that the microgels’ formation is also affected by the viscosity of the cured mixture.

**Figure 11:**
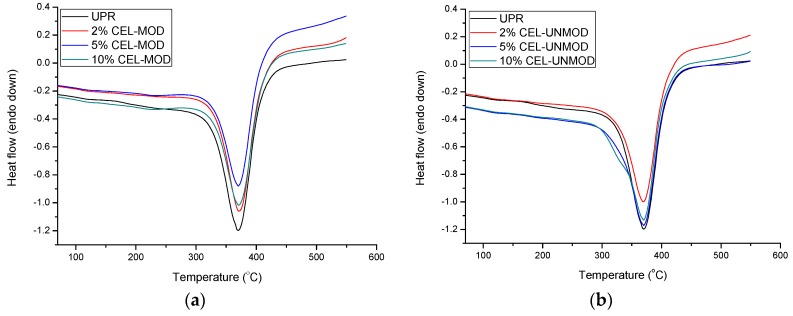
Differential scanning calorimetry (DSC) curves of the UPR and composites with CEL-MOD and CEL-UNMOD. (**a**) CEL-MOD; (**b**) CEL-UNMOD.

The obtained composites were also investigated by differential scanning calorimetry (DSC). The DSC curves are shown in Figure 11. The values of maximum decomposition (T_d_) and enthalpy of decomposition (ΔH_d_) are listed in Table 2. The thermal behaviour of the composites is characterized by a well-shaped calorimetric profile containing a single symmetric peak in the range of 300–450 °C. It corresponds to the pyrolytic degradation observed in the TG/DTG study. Information about the caloric effect associated with this process, which is the result of the overlapping of both endo- and exothermic effects, has allowed to confirm the impact of MCC modification on the properties of the obtained materials. The highest value of decomposition enthalpy among composites with CEL-UNMOD was obtained for the system containing 5% filler (Figure 11). The increased share of endothermic effects confirms the largest heterogeneity of the systems with 5% and 10% of CEL-UNMOD and their two-stage distribution noted in the TG/DTG tests. This is because of the formation of a protective layer derived primarily from the pyrolysis of the matrix. T_d_ may also indicate the quality of the dispersion of the filler in the matrix. The use of CEL-MOD in composites increases T_d_, while the use of CEL-UNMOD reduces their value compared with UPR. Additionally, no exothermic effect associated with the post-curing process was observed, which confirms complete cross-linking of the samples.

## 4. Conclusions

Composites of unsaturated polyester resin with the MCC modified with methacrylic anhydride and, comparatively, those with the unmodified MCC were obtained. The presence of methacrylic groups on the MCC surface improved mechanical properties of the composites compared with the systems with the unmodified MCC. The values of flexural strength and strain at break increased. This resulted from the improvement of the filler miscibility in the resin and limiting its ability to form agglomerates. However, MCC modification resulted in a reduction of intermolecular hydrogen bond interactions between matrix (polyester) carbonyl groups and filler hydroxyl groups. This resulted in a smaller increase in flexural modulus, especially visible in systems containing 10% of filler. The improvement of CEL-MOD mixtures homogeneity also affected their thermal stability, which is particularly visible in the case of 5% and 10% of the CEL-UNMOD systems characterized by decomposition in two temperature ranges. At the same time, modification reduced the friction of the matrix with the filler, thus affecting the composites’ rigidity. In addition, the presence of unsaturated bonds in the filler structure allowed it to be incorporated into the structure of the polymer matrix formed during curing. As a result, the composite containing 5% of the modified MCC had practically identical damping values and glass transition temperature as the 2% CEL-UNMOD system.

## Figures and Tables

**Table 1:** Mechanical and thermomechanical data of samples.

Properties	UPR	COPOLYMERS					
		CEL-MOD			CEL-UNMOD		
		2%	5%	10%	2%	5%	10%
Flexural modulus/GPa	3.00 ± 003	2.99 ± 003	3.11 ± 0.04	3.08 ± 0.07	3.04 ± 0.25	2.95 ± 0.11	3.25 ± 0.02
Flexural strength/MPa	81 ± 2.1	76.7 ± 2.6	74.8 ± 4.4	73.3 ± 4.6	59.7 ± 5.5	57.8 ± 1.7	53 ± 2.3
Strain at break/%	2.93 ± 0.12	2.72 ± 0.14	2.58 ± 0.2	2.49 ± 0.18	2.02 ± 0.16	2.02 ± 0.12	1.68 ± 0.08
Hardness/MPa ^a^	128.1	129.7	130.4	134.4	127.3	126.5	129.7
E’(25 °C)/GPa	2.81	2.82	2.81	2.99	2.82	2.86	2.99
E’(180 °C)/MPa	95.6	89.1	93.6	117.3	96.7	89.7	117
T_g_/°C ^b^	141.9	143.2	143.3	141.4	143.5	139.9	141
tg δ_max_	0.206	0.195	0.215	0.174	0.216	0.206	0.198
FWHM/°C ^c^	49.4	49.4	49.2	50.2	48	50.8	49

^a^ Brinell method; ^b^ Determined from tg δ_max_; ^c^ FWHM, full width at half maximum. CEL-MOD, modified microcrystalline cellulose (MCC); CEL-UNMOD, unmodified MCC.

**Table 2:** Thermograms (TG/DTG) and differential scanning calorimetry (DSC) data.

Properties	UPR	CEL-MOD	CEL-UNMOD	COPOLYMERS					
				CEL-MOD			CEL-UNMOD		
				2%	5%	10%	2%	5%	10%
T_max1_ (°C)	375.4	356.6	340.5	375.9	376.7	377.2	375.5	367.7	372.5
T_max2_ (°C)	-	-	-	-	-	-	-	568.2	595.2
Mass change in the range 220–300 °C (%)	3.49	8.60	1.71	3.36	3.66	4.00	3.35	4.98	4.07
Sum of changes in mass of individual components of composites in the range 220–300 °C (%)	-	-	-	3.59	3.75	4.00	3.45	3.40	3.30
The difference in the mass change of the composites and their individual components (%)	-	-	-	−0.23	−0.09	0	−0.10	1.58	0.77
T_d_ (°C)	369.7	-	-	371.2	369.8	370.6	369.0	369.4	369.3
ΔH_d_ (J/g)	306.2	-	-	293.5	260.6	276.9	269.8	285.0	280.4
